# A Three-Pool Model Dissecting Readily Releasable Pool Replenishment at the Calyx of Held

**DOI:** 10.1038/srep09517

**Published:** 2015-03-31

**Authors:** Jun Guo, Jian-long Ge, Mei Hao, Zhi-cheng Sun, Xin-sheng Wu, Jian-bing Zhu, Wei Wang, Pan-tong Yao, Wei Lin, Lei Xue

**Affiliations:** 1 State Key Laboratory of Genetic Engineering, Collaborative Innovation Center of Genetics and Development, Department of Physiology and Biophysics, School of Life Sciences, Fudan University, Shanghai, P.R. China, 200433; 2 Synaptic Transmission Section, National Institute of Neurological Disorders and Stroke, Bethesda, Maryland, 20892; 3 School of Mathematical Sciences, Centre for Computational Systems Biology and Shanghai Centre for Mathematical Sciences, Fudan University, P.R.China, 200433

## Abstract

Although vesicle replenishment is critical in maintaining exo-endocytosis recycling, the underlying mechanisms are not well understood. Previous studies have shown that both rapid and slow endocytosis recycle into a very large recycling pool instead of within the readily releasable pool (RRP), and the time course of RRP replenishment is slowed down by more intense stimulation. This finding contradicts the calcium/calmodulin-dependence of RRP replenishment. Here we address this issue and report a three-pool model for RRP replenishment at a central synapse. Both rapid and slow endocytosis provide vesicles to a large reserve pool (RP) ~42.3 times the RRP size. When moving from the RP to the RRP, vesicles entered an intermediate pool (IP) ~2.7 times the RRP size with slow RP-IP kinetics and fast IP-RRP kinetics, which was responsible for the well-established slow and rapid components of RRP replenishment. Depletion of the IP caused the slower RRP replenishment observed after intense stimulation. These results establish, for the first time, a realistic cycling model with all parameters measured, revealing the contribution of each cycling step in synaptic transmission. The results call for modification of the current view of the vesicle recycling steps and their roles.

Repetitive firing causes short-term depression (STD) at many synapses^1^, which plays an important computational role in neuronal circuits^2^. A major mechanism underlying STD during intense stimulation is depletion of the readily releasable pool (RRP)^1^. The degree of RRP depletion and its subsequent replenishment may determine the degree and the time course of STD, and thus the synaptic strength and neuronal circuit function^1^. Given such important roles, however, the source of vesicles that are mobilised to replenish the RRP and the mechanism that determines the rate of the RRP replenishment remain poorly understood.

Studies several decades ago revealed that intense stimulation slows the recovery of STD^3^. Accordingly, a vesicle cycling model composed of two pools, the RRP and the reserve pool (RP: containing all vesicles except those in the RRP) was proposed, in which fused vesicles are retrieved via endocytosis to the RP, which then supplies vesicles to the RRP. Depletion of the RP could thus account for the slower recovery of STD^1^,^3^. Consistent with this model, blocking endocytosis at the neuromuscular junction of *shibire* mutants led to a progressive decline in transmitter release during repetitive stimulation^4^,^5^. However, this model has not been tested rigorously by measuring all model parameters, including pool sizes, RP-RRP kinetics, and endocytosis rates, and by determining whether the model with measured parameters matches the observed STD. More importantly, many additional factors have been reported since this model was proposed, including various forms of endocytosis, vesicle pools, and various rates of the RRP replenishment, which necessitates a rethinking the model.

Studies in the last 15 years have revealed that endocytosis can be rapid (1–2 s) or slow (10–30 s)^6^,^7^. Rapid endocytosis is often considered to be kiss-and-run fusion and retrieval^8^, which involves rapid fusion pore opening and closure at the same site^9^,^10^,^11^,^12^. It has been proposed that kiss-and-run locally recycles vesicles within the RRP at cultured hippocampal synapses^13^,^14^,^15^, though whether rapid kiss-and-run exists at this synapse is still debated^8^.

Slow endocytosis, mediated by a clathrin-dependent mechanism^16^,^17^, is considered a major endocytic pathway^17^. This pathway is hypothesised to retrieve vesicles into a small recycling pool^18^. The concept of a small recycling pool was based on the finding that only a small fraction (~5–20%) of vesicles can be stained with the styryl dye (e.g., FM1-43) during low and intermediate frequency stimulation that releases all recycling vesicles^18^. If this concept holds, two predictions can be made: 1) the majority of vesicles residing in the RP are irrelevant to synaptic transmission under many physiological conditions, and 2) interference of this small recycling pool will have a crucial and rapid influence on the RRP replenishment. However, our previous studies challenged the presence of a small recycling pool. First, by simultaneously recording presynaptic capacitance and postsynaptic EPSC in the absence or presence of glutamate at rat calyces, we suggest a large recycling pool ~46 times the size of RRP, which is close to the total vesicle amount measured by electron microscopy^19^,^20^. Such a large recycling pool confirms that almost all vesicles are mobilised to maintain synaptic transmission upon high frequency stimulation^20^. Second, we have shown that blocking of both rapid and slow endocytosis does not affect the rate of RRP replenishment^21^, which rules out the possibility that endocytosed vesicles recycle within the RRP or a small recycling pool because it predicts slower RRP replenishment when endocytosis is blocked. Therefore, our previous results suggest that endocytosed vesicles are retrieved into a large recycling pool instead of a small one.

The RRP replenishment time course is often bi-exponential with a calcium/calmodulin-dependent rapid component of ~1 s or less^22^,^23^,^24^,^25^. However, the source of vesicles responsible for rapid and slow RRP replenishment is unclear. Original candidates that were considered include vesicles formed by rapid endocytosis and the small recycling pool^18^. However, both candidates have been ruled out based on our previous studies^20^,^21^. Furthermore, we found that the RRP replenishment time course was slowed down by more intense stimulation^21^. Since the RRP replenishment is calcium/calmodulin-dependent, stronger stimulation should increase the intracellular calcium concentration, and facilitate calmodulin function to speed up the RRP replenishment^23^,^25^. Thus, a mechanism other than calcium/calmodulin regulation must be involved.

Here, we provide a realistic three-pool model with all parameters experimentally measured, which shows the contribution of each step in exo- and endocytosis, and explains why the RRP replenishment is composed of rapid and slow components, and why the RRP replenishment could be slowed down by intense stimulation. We further applied this model to evaluate the contribution of endocytosis under prolonged action potential-like stimulation train. The current view of vesicle cycling may need to be modified to include the large recycling pool^20^ and the new intermediate pool in order to accommodate these new findings.

## Methods

### Slice preparation and electrophysiology

Slice preparation was similar as described previously^7^,^26^,^27^. Briefly, Postnatal day 7–10 (p7–p10) old Wistar rats of either sex were decapitated and the brain stem slices of ~200 μm thick containing the medial nucleus of the trapezoid body (MNTB) were prepared using a vibratome (VT 1200s, Leica, Germany). Recordings were made at room temperature (22–24°C). Whole-cell capacitance measurements were made with the EPC-10 amplifier together with the software lock-in amplifier (PatchMaster, HEKA, Lambrecht, Germany). Exocytosis and endocytosis are represented by capacitance changes after conditioning stimulation^20^,^27^. Measurements of the RRP size and RRP replenishment time course were similar to previous reports^21^,^26^. The presynaptic pipette (3.5–5 MΩ) solution contained (in mM): 125 Cs-gluconate, 20 CsCl, 4 MgATP, 10 Na_2_-phosphocreatine, 0.3 GTP, 10 HEPES, 0.05 BAPTA (pH 7.2, adjusted with CsOH). Measurements of the AMPA receptor-mediated EPSC were made by whole-cell patch at the postsynaptic principle neurons. The postsynaptic pipette (2.5–4 MΩ) solution contained (in mM): 125 K-gluconate, 20 KCl, 4 Mg-ATP, 10 Na_2_-phosphocreatine, 0.3 GTP, 10 HEPES, and 0.5 EGTA (pH 7.2, adjusted with KOH). The series resistance (<10 MΩ) was compensated by 90% (lag 10 μs). For recordings of the EPSC, kynurenic acid (1 mM) was added in the bath solution to relieve saturation and desensitisation of postsynaptic AMPA receptors. The holding potential was –80 mV for both presynaptic and postsynaptic recordings if not mentioned. Statistical analysis used a t test unless otherwise noted, and means are presented as ± SE. All the methods were carried out in accordance with the approved guidelines and all animal experimental protocols were approved by the Animal Care and Use Committee of Fudan University.

### Three-pool vesicle cycling model

With a large recycling pool ~46 times the size of RRP, we could not find any existing two-pool model that can fit the bi-exponential RRP replenishment with the rapid component being reduced by more intense stimulus^20^,^21^. We proposed a new model composed of three vesicle pools, a reserve pool (RP), an intermediate pool (IP) between the RP and the RRP, and a RRP with reversible first order kinetics between RP and IP (k_2_, k_-2_) and between the IP and RRP (k_1_, k_-1_). The kinetic scheme for these three pools is: 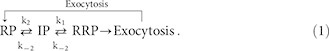
Endocytosed vesicles enter only the RP, not the IP or RRP^21^, and the percentage of rapid and slow endocytosis was from our previous study^27^. As blocking endocytosis does not affect RRP replenishment^21^, to simplify the mathematical reasoning, we did not include the endocytosed vesicles when calculating all of the parameters and included them back using MATLAB Simbiology toolbox (v2014a, the Mathworks, USA) to generate the vesicle cycling in each time step. The simplified scheme is shown below:
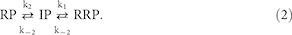


This model could explain the bi-exponential time course of replenishment by assuming a rapid rate constant for k_1_ and k_-1_, a slow rate constant for k_2_ and k_-2_, and a small IP size. The small IP could be depleted more by a more intense stimulus, explaining why the replenishment_rapid_ amplitude was decreased, instead of increased, after more intense stimulation. In the following, we performed quantitative calculations to determine the parameters (initial size of IP, RP, and k_1_, k_-1_, k_2_, and k_-2_) that fit our data.

From scheme 2,
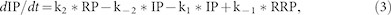
where RP, IP, and RRP are the corresponding amount at time t after stimulation. 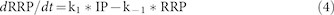
From the law of conservation of mass,
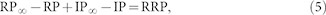
where ∞ denotes the steady state, or the resting condition. (We assumed them the same here to simplify calculation.)

Based on equations 3–5, we obtained the second order differential equation of RRP:
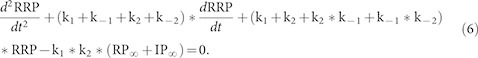


The solution of this differential equation is: 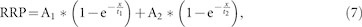
where
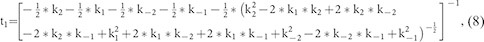

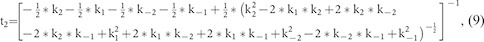

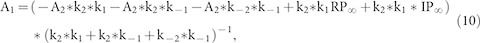
and A_2_ is a constant (the integral constant).

**Figure 1:**
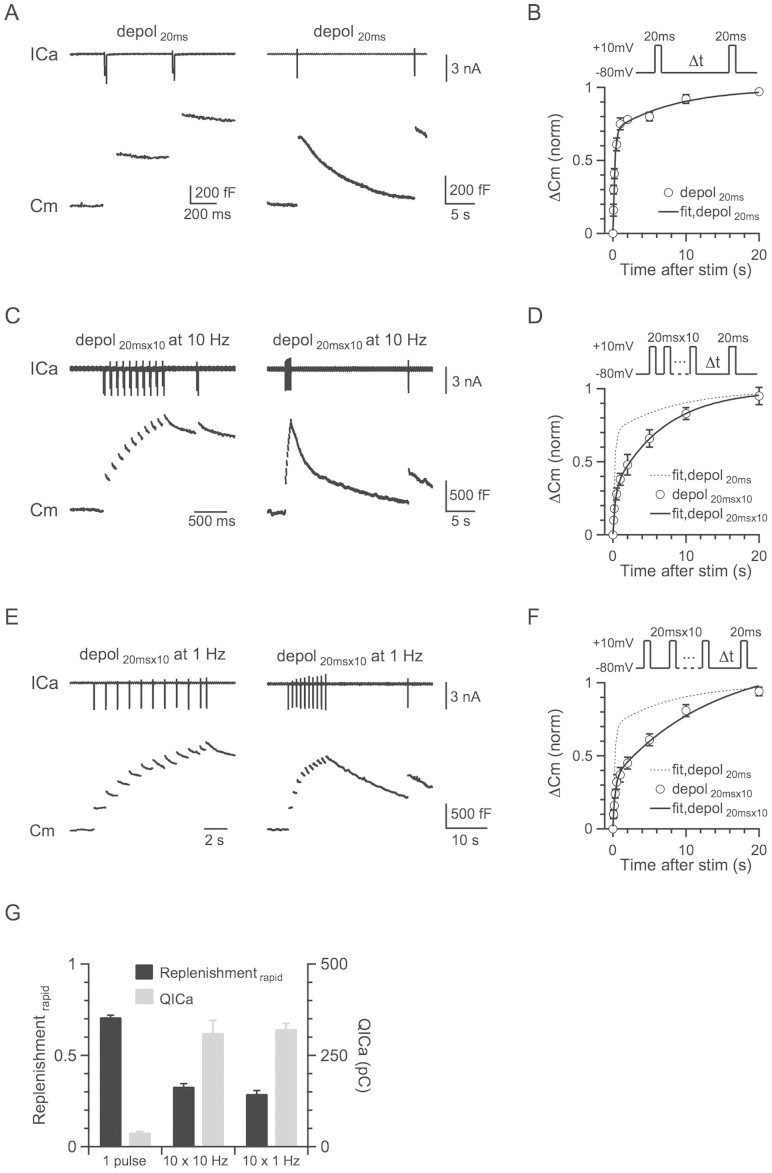
More intense stimulation slows the RRP replenishment. (A) Left: Sampled presynaptic calcium current (ICa, upper) and membrane capacitance (Cm, lower) induced by a 20 ms depolarisation followed by a conditioning pulse of 20 ms depolarization with a 0.5 s interval. Right: Similar to Left, except that the stimulus interval is 20 s. (B) Upper: The protocol used to measure the RRP replenishment after a 20 ms depolarisation pulse. Lower: Cm induced by a 20 ms depolarisation applied at various intervals after the conditioning stimulus (n = 8). Data were normalised to the Cm induced by the conditioning pulse, and fit with a bi-exponential function (solid line) where A_1_ = 0.71, τ_1_ = 0.26 s, A_2_ = 0.29, τ_2_ = 9.5 s. (C) Similar to A, except that the conditioning stimulus was 10 pulses of 20 ms depolarisation at 10 Hz. (D) Similar to B, except that the conditioning stimulus was 10 pulses of 20 ms depolarisation at 10 Hz (n = 11). Data were normalised to the Cm induced by a 20 ms depolarisation applied at >30 s after the conditioning stimulus, and fit with a bi-exponential function (solid line) where A_1_ = 0.33, τ_1_ = 0.38 s, A_2_ = 0.67, τ_2_ = 7.8 s. The fitting curve of single pulse was also plotted for comparison (dotted line). (E) Similar to A, except that the conditioning stimulus was 10 pulses of 20 ms depolarisation at 1 Hz. (F) Similar to D, except that the conditioning stimulus was 10 pulses of 20 ms depolarisation at 1 Hz (n = 6). Data were fit with a bi-exponential function where A_1_ = 0.29, τ_1_ = 0.25 s, A_2_ = 0.71, τ_2_ = 7.9 s. (G) The plot of the normalised RRP replenishment_rapid_ amplitude versus calcium influx (QICa) in a 20 ms depolarisation pulse and 10 pulses of 20 ms depolarisation at 1–10 Hz (QICa: 38.9 ± 2.6 pC, n = 8, single pulse; 312 ± 34 pC, n = 11, 10 pulses at 10 Hz; 323 ± 15 pC, n = 6, 10 pulses at 1 Hz).

Based on equation 7, at the steady state (t = ∞), RRP_∞_ = A_1_ + A_2_. For simplicity, we normalised the RRP_∞_ to 1. Thus,
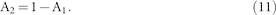


Equation 7 indicates that the RRP replenishment time course is bi-exponential. Equations 8–9 indicate that the time constants for both rapid and slow RRP are independent of the stimulation intensity but depend on only four kinetic constants, k_1_, k_−1_, k_2_, and k_−2_. These features are consistent with our experimental result that the RRP replenishment time course is bi-exponential, and that the time constants for rapid and slow RRP replenishment did not change significantly after 1–10 pulses of 20 ms depolarisation (Fig. 1). Such a consistency further strengthened our model.

In the steady state (t = ∞), or resting conditions, 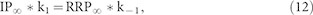

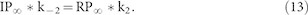
Because the recycling pool, which included RRP_∞_, IP_∞_, and RP_∞_, was 46 times the size of RRP_∞_^20^, where RRP_∞_, IP_∞_, and RP_∞_ were all normalised to RRP_∞_ (RRP_∞_ = 1). Thus, RP_∞_ = 45 − IP_∞_. These calculations allow us to rewrite equations 12 and 13 as equations 14 and 15: 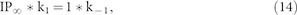

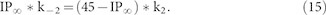


In equations 8, 9, 14, and 15, t_1_ and t_2_ (the time constant for rapid and slow RRP replenishment) could be obtained by fitting experimental measurements after a single 20 ms depolarisation with equation 1 (0.26 and 9.5 s, Fig. 1A). The normalised RRP_∞_ was 1, and the normalised RP_∞_ = 45 - IP_∞_ (RRP_∞_ + IP_∞_ + RP_∞_ = 46). Thus, there are five unknown parameters, k_1_, k_-1_, k_2_, k_-2_, and IP_∞_ in four equations (8, 9, 14, 15), which do not allow us to solve these five parameters.

To solve these five parameters, we varied the IP_∞_ value from 0.5 to 10 with an incremental step of 0.1. For each of these IP_∞_ values, we obtained a set of rate constants (k_1_, k_-1_, k_2_, and k_-2_) from equations 8, 9, 14, and 15. With each of these IP_∞_ values and their corresponding rate constants (k_1_, k_-1_, k_2_, and k_-2_), we numerically calculated the IP, RP, and RRP changes as a function of time after depletion of the RRP by a single 20 ms depolarisation. The numerical calculation was based on equations 3–4 with a Δt of 0.1 ms or less using MATLAB Simbiology toolbox (v2014a, the Mathworks, USA). The calculated RRP replenishment time course was compared to the experimentally measured time course after a 20 ms depolarisation using the Kolmogorov-Smirnov test and the least-squares test. The best fitting group for the parameters is as follows: IP_∞_ = 2.7, RP = 42.3, k_1_ = 0.8892, k_-1_ = 2.4008, k_2_ = 0.0093, and k_-2_ = 0.1546 (unit: sec^-1^).

## Results

### The rate of the RRP replenishment was slower after more intense stimulation

Many previous studies confirmed that a 10–20 ms depolarisation from −80 to +10 mV depletes the RRP at the calyx of Held^7^,^26^,^28^,^29^. At various times (Δt = 0.05–20 s) after a conditioning 20 ms depolarisation (−80 to +10 mV, if not mentioned), which depleted the RRP (459 ± 29 fF, n = 11), we applied a 20 ms depolarisation to measure the resulting capacitance jump (ΔCm), which reflected the recovery of the RRP (Fig. 1A). We also used different intensities of conditioning stimuli, including a single 20 ms depolarisation, and 10 pulses of 20 ms depolarisation delivered at 10 or 1 Hz (Figs. 1C, E). After a 20 ms depolarisation, the RRP recovery could be fitted with a bi-exponential function (ΔCm = A_1_*[1 − exp(−t/τ_1_)] + A_2_*[1 − exp(−t/τ_2_)]) where A_1_ = 0.71, τ_1_ = 0.26 s, A_2_ = 0.29, τ_2_ = 9.5 s (Fig. 1B), which was similar to previous reports^24^,^25^,^26^ (data in Fig. 1 were adopted from our previous study^21^).

After 10 pulses of 20 ms depolarisation at 10 Hz, which induced a capacitance jump of 1260 ± 72 fF (n = 11), the RRP replenishment could also fit a bi-exponential function with A_1_ = 0.33, τ_1_ = 0.38 s, A_2_ = 0.67, τ_2_ = 7.8 s (Fig. 1D). Compared to the fitted replenishment curve after a 20 ms depolarisation (dotted curve in Fig. 1D, same as Fig. 1B), the rapid component of replenishment (replenishment_rapid_) decreased, whereas the slow component of replenishment (replenishment_slow_) increased. Similarly, after 10 pulses of 20 ms depolarisation at 1 Hz, the RRP replenishment also slowed down (Fig. 1F, A_1_ = 0.29, τ_1_ = 0.25 s, A_2_ = 0.71, τ_2_ = 7.9 s). Our results contradict previous studies that suggested a calcium/calmodulin-dependent mechanism^23^,^25^. The total calcium charge dramatically increased from a single 20 ms depolarisation pulse to 10 depolarisation pulses of 1 or 10 Hz, but the RRP replenishment slowed down instead of speeding up, which could not be explained by the up-regulation of calmodulin function (Fig. 1G). Furthermore, it is also very interesting that the time constant of the RRP replenishment was very similar among different stimulation protocols (Figs. 1B, D and F).

### Rapid and slow vesicle traffic among three pools underlie rapid and slow RRP replenishment

Our observation that the replenishment_rapid_ amplitude was significantly reduced when calcium charge (QICa) was increased by ~8 times in Fig. 1G could be resolved if 10 depolarising pulses depleted most of the recycling pool that provided vesicles to the RRP. However, we have shown that the recycling pool was ~46 times the size of RRP^20^, and it was only slightly reduced by 10 depolarising pulses at 1 or 10 Hz, which only released vesicles equivalent to ~3–6 times the size of RRP. This slight reduction of the recycling pool could not account for the significant decrease in the replenishment_rapid_ amplitude. Therefore, we proposed a new three-pool model to account for this phenomenon (see Methods for details).

From scheme (1), the RRP size can be derived as a function of time after depletion of the RRP. The analytical solution of the RRP (equation 7) is a bi-exponential function, which is consistent with our experimental observation (Figs. 1D, F). Based on the previously measured recycling pool size, which was the sum of the three pools (RRP_∞_ + IP_∞_ + RP_∞_ = 46, where ∞ denotes the resting condition and all parameters are normalised to RRP_∞_), we found that the numerically calculated RRP replenishment time course after a 20 ms depolarisation best fit the observed data (Fig. 1B) when IP_∞_ = 2.7, RP_∞_ = 42.3 (RRP_∞_ was normalised to 1), k_1_ = 0.8892, k_−1_ = 2.4008, k_2_ = 0.0093, and k_−2_ = 0.1546 sec^−1^ (calculated from equations 8–9 and 14–15).

**Figure 2:**
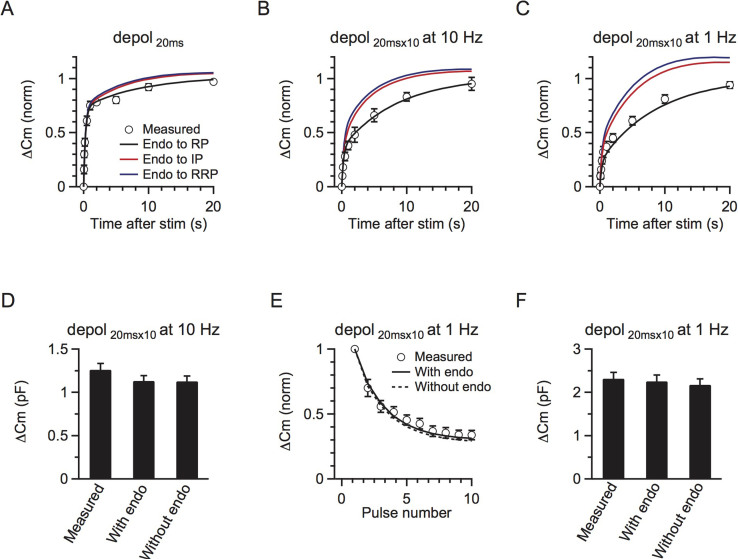
A three-pool model underlies rapid and slow RRP replenishment. (A) The model-predicted RRP replenishment curves with endocytosed vesicles recycling to RP (black), IP (red) and RRP (blue) after a 20 ms depolarisation pulse. The measured data are also plotted for comparison (circle, same as Fig. 1B). The legend also applies to B and C. (B) Similar to A, but with a conditioning stimulus of 10 pulses of 20 ms depolarisation at 10 Hz. (C) Similar to A and B, but with a conditioning stimulus of 10 pulses of 20 ms depolarisation at 1 Hz. (D) The total measured and predicted ΔCm with and without endocytosis induced by 10 pulses of 20 ms depolarisation at 10 Hz (n = 11). (E) The model-predicted (with endocytosis: black curve, without endocytosis: dotted curve) and the measured (circle) ΔCm induced by each depolarising pulse (20 ms depolarisation) during a 10-pulse train at 1 Hz. (F) Similar to D, except that the conditioning stimulus was 10 pulses of 20 ms depolarisation at 1 Hz (n = 6).

The above parameters were obtained by comparing the model with the observed RRP replenishment after a single 20 ms depolarisation (Fig. 2A, black curve). To further determine whether these parameters were appropriate, we used the model with these parameters to generate several predictions that were not related to the single 20 ms depolarisation data. The prediction was made by numerical calculation of the RRP replenishment using equations 3–4.

First, the model-predicted time course of the RRP replenishment after 10 pulses of 20 ms depolarisation at 10 Hz matched well with the observed time course (Fig. 2B, black curve, p = 0.93, K-S test). The predicted time course was fitted by a bi-exponential equation with parameters (A_1_ = 0.37, τ_1_ = 0.3 s, A_2_ = 0.63, τ_2_ = 8.3 s, Fig. 2B, black curve) similar to those obtained from fitting the observed data (Fig. 1D). Second, the model-predicted RRP replenishment matched well with the measured one after 10 depolarising pulses at 1 Hz (Fig. 2C, black curve, p = 0.90, K-S test). The model-predicted parameters were also very close to those in observed ones (A_1_ = 0.22, τ_1_ = 0.2 s, A_2_ = 0.78, τ_2_ = 6.5 s). Third, the model-generated exocytosis during each time step matched well with the experimental results. As the model was derived from the RRP replenishment data, it would be supportive if the model could also predict exocytosis. For 10 depolarising pulses at 10 Hz, it is difficult to accurately measured the capacitance jump after each stimulus^27^, so we only compared the total capacitance jump. The model predicted a total ΔCm of 2.46 times the RRP evoked by 10 depolarising pulses at 10 Hz. By multiplying this value with the capacitance jump induced by a 20 ms depolarisation, which was the RRP size, it predicted a ΔCm of 1131 ± 61 fF (1127 ± 61 fF if endocytosed vesicles are not included, n = 11), which closely matched the measured ΔCm after 10 depolarising pulses at 10 Hz (1260 ± 72 fF, n = 11, p = 0.2, Fig. 2D). For 10 depolarising pulses at 1 Hz, we could accurately compare the capacitance jump after each stimulus. The model predicted a gradual decrease of the capacitance jump induced by each stimulus during 10 depolarising pulses at 1 Hz, which matched the measurement well (p = 0.7 with endocytosis, K-S test, Fig. 2E). The predicted total exocytosis amount (with endo: 2.3 ± 0.1 pF, without endo: 2.2 ± 0.1, n = 6) also closely matched the measured net exocytosis (2.3 ± 0.1 pF, n = 6, p = 0.8 with endocytosis, Fig. 2F). All these matches between predictions and experimental results (Figs. 2B–F) further strengthened our model with parameters described above.

The fast IP-RRP kinetics (k_1_ = 0.8892, k_−1_ = 2.4008) and the slow RP-IP kinetics (k_2_ = 0.0093, k_−2_ = 0.1546) explained why replenishment was bi-exponential with rapid (τ_1_) and slow (τ_2_) time constants being controlled mostly by k_1_ and k_−1_, and k_2_ and k_−2_, respectively. Our numerical solution to τ_1_ and τ_2_ also further illustrated why the time constants in different stimulation protocols are roughly the same (equations 8–9). Our numerical calculation (equations 3–4) also showed that the IP size was 2.7 immediately after a 20 ms depolarisation, but decreased to ~0.9–1.4 immediately after 10 pulses of depolarisation at 1–10 Hz. The IP size after stimulation controlled the replenishment_rapid_ amplitude, which explains why the replenishment_rapid_ component was reduced after 10 pulses of depolarisation at 1–10 Hz.

High temperatures have been shown to lead to accelerated endocytosis^30^ and faster recovery from synaptic depression at calyces^31^. Recently, ultra-fast endocytosis was observed at hippocampal neurons at 34°C^32^. To test whether our model could also be applied at physiological temperatures, we performed similar experiments at a more physiological temperature (~34°C). At higher temperatures, RRP replenishment was dramatically accelerated after a 20 ms depolarisation or 10 pulses of 20 ms depolarisation at 1–10 Hz (Figs. S1, see Supplementary Information I for details) and still fit our model well if we multiplied all of the rate constants by a ratio of 2–4.

### Neither slow nor rapid endocytosis recycles vesicles within a small recycling pool

We previously showed that both rapid and slow endocytosis do not recycle vesicles within the RRP^21^. The RRP replenishment showed no difference after blocking endocytosis with GTPγS, which ruled out the possibility of a small recycling pool. We further used the current model to verify this conclusion.

**Figure 3:**
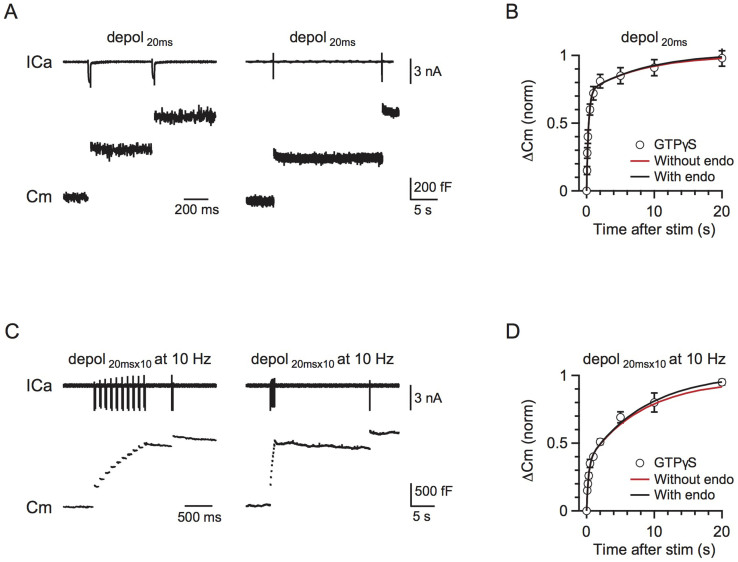
Rapid and slow endocytosis do not recycle vesicles in a small recycling pool. (A) Left: Sampled presynaptic calcium current (ICa, upper) and membrane capacitance (Cm, lower) induced by a 20 ms depolarisation applied at 0.5 s after a conditioning pulse of 20 ms depolarization with 0.3 mM GTPγS in place of GTP in the pipette solution. Right: Similar to Left, except that the stimulus interval is 20 s. (B) The model-predicted RRP replenishment curves with (scheme 1, black) and without endocytosis (scheme 2, red) after a 20 ms depolarisation pulse. Data measured with 0.3 mM GTPγS in the pipette solution are also plotted for comparison (circle). (C) Similar to A, but with a conditioning stimulus of 10 pulses of 20 ms depolarisation at 10 Hz. (D) Similar to B, except that the conditioning stimulus was 10 pulses of 20 ms depolarisation at 10 Hz.

In scheme (1), we compared the influence of endocytosis in three circumstances: 1) endocytosed vesicles directly recycle within the RRP, 2) endocytosed vesicles enter the IP, and 3) the endocytosed vesicles enter the RP. Endocytosed vesicles did not directly enter the RRP because blocking endocytosis did not affect RRP replenishment (Fig. 3). Endocytosed vesicles did not enter the IP because the recycling pool size was 46 times the size of RRP^20^, whereas the IP_∞_ size was only 2.7. Furthermore, a significant amount of endocytosis occurred during 10 pulses of 20 ms depolarisation at 1 Hz^27^. If these endocytosed vesicles directly enter the IP, RRP replenishment should be accelerated (Fig. 2C). In contrast, RRP replenishment was slower compared to a single 20 ms depolarisation (Figs. 1D, F). Our model also confirmed that, if the endocytosed vesicles entered the RRP or IP, RRP replenishment would be accelerated, which was not consistent with the experimental observations (Figs. 2A–C). Therefore, we conclude that endocytosed vesicles enter the RP before being mobilised to the IP.

### The impact of endocytosis on synaptic transmission

**Figure 4:**
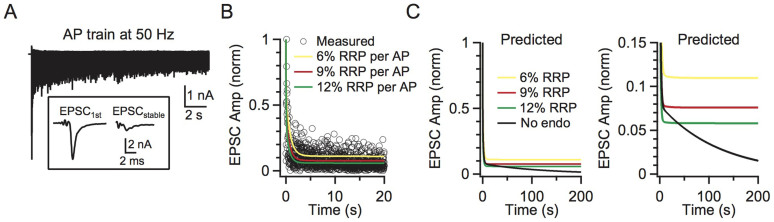
Impact of endocytosis during high frequency stimulation. (A) Sampled trace of EPSC recordings during a 50 Hz action potential train. Inset shows the large initial EPSC and the small stabilised EPSC for comparison. (B) The model-predicted (curves) and the measured (n = 4, circle) amplitudes of the EPSCs during a 50 Hz action potential train. Predicted traces for different depleting percentages after a single action potential are shown in different colours (yellow: 6%, red: 9%, green: 12%). Data were normalised to the first response. Each circle represents the mean amplitude from four synapses (for clarity, s.e.m. is not included). Experimental data were collected from horizontal brain slices, where a bipolar electrode was positioned in the midline of the trapezoid body to induce presynaptic action potentials and thus EPSCs^54^. The model included endocytosis (scheme 1). (C) The model-predicted EPSC amplitude during action potential stimulation at 50 Hz with (scheme 1, colours meanings are the same as B) and without (scheme 2, black) endocytosis. Left and right panels show the same data at different scales.

Endocytosis not affecting RRP replenishment raises doubts about the role of endocytosis. To determine the impact of endocytosis during repetitive firing, we mimicked the physiological firing frequency of 50 Hz by depleting the RRP by ~6–12% for each action potential in our model. We used 50 Hz because the calyx may fire spontaneously at a mean rate of ~50–60 Hz in vivo^33^. We used 6–12% because an action potential may deplete ~6% of the RRP^34^,^35^ and the release probability may be facilitated by ~30–100% during stimulation^36^,^37^. Based on our previous study^27^, we assumed that ~70% of fused vesicles were retrieved rapidly with a time constant of ~1.5 s, and the remaining 30% were retrieved slowly with a time constant of ~15 s. With scheme (1), the model predicted that the release evoked by a single action potential reached a steady state of 6–11% of the first response (Fig. 4B), which was similar to the steady-state reduction of the EPSCs evoked by axonal fibre stimulation at 50 Hz in the presence of 1 mM kynurenic acid, which prevents postsynaptic AMPA receptor saturation (Figs. 4A–B, n = 4 synapses).

In the absence of endocytosis, the model (scheme 2) predicted a gradual decline in release gradually to 0 (Fig. 4C). Compared to the prediction in the presence of endocytosis (scheme 1), the decline was obvious only after stimulation for > 10 s at 50 Hz (Fig. 4C, right). This effect was not trivial to the calyx of Held synapse. The calyx fires spontaneously at a mean rate of ~50 Hz in vivo and, without any previous firing, an action potential releases several hundred vesicles that ensure firing of a postsynaptic action potential^33^. With firing at 50 Hz, the release induced by an action potential decreases to ~6–11% (Fig. 4B), which is still sufficient to induce a postsynaptic action potential^38^. Without endocytosis, release will eventually decrease to 0 (Fig. 4C), which abolishes postsynaptic firing. Thus, endocytosis is essential for maintaining transmitter release and synaptic transmission during high frequency firing. We could not confirm this suggestion experimentally by blocking endocytosis with GTPγS, because a GTP-independent endocytosis develops during strong stimulation^39^. However, the gradual decrease in release when endocytosis is blocked in *shibire* mutants is consistent with our suggestion^4^,^5^,^40^.

## Discussion

Based on our previous finding that both rapid and slow endocytosis recycled vesicles to a large recycling pool instead of within the RRP or a small recycling pool, we proposed a model composed of three pools, a large RP ~42.3 times the size of RRP, a RRP and a small IP ~2.7 times the size of RRP in between^20^. We found that rapid IP-RRP kinetics, slow RP-IP kinetics, and limited IP size are responsible for the rapid and slow components of RRP replenishment, and the slower RRP replenishment with more intense stimulation, as observed at many synapses^13^,^22^,^35^,^41^,^42^. This realistic model reveals the contribution of each vesicle cycling step in the maintenance of synaptic transmission, and thus, in the generation and recovery of STD during repetitive stimulation.

There are other explanations for the two components of RRP replenishment. Garcia-Perez et al. reported similar RRP replenishment at hippocampal synapses and provided “delayed depression” as an explanation, which lacked experimental verification^43^. Otsu et al. proposed two general models that accounted for the fast and slow time constants of RRP replenishment^42^. However, neither model could explain why blocking endocytosis does not affect RRP replenishment. First, our previous results ruled out the possibility that fused vesicles can recycle within the RRP^21^. Second, the relatively small RP size (8–12 times the RRP), which could be dramatically decreased during STD (more than 50% decrease^42^), and the fast docking/undocking kinetics between RP and RRP suggest a significant role of endocytosis in RRP replenishment, but this is also ruled out by our results. Pyle et al. proposed a similar two-pool model, suggesting that the RRP could be refilled by either rapid retrieval within the RRP or new recruitment from the reserve pool at the hippocampus^13^, which still contradicts our findings^21^. A recent study showed that exocytosed vesicles are not generally reused within 40 s, which further strengthens our conclusion that rapid reuse may not contribute to rapid RRP replenishment^44^. With all parameters experimentally measured, our model is more quantitatively accurate and more capable of explaining RRP replenishment after different stimulations.

The rapid component of the RRP replenishment time course has been hypothesised to be caused by a calcium/calmodulin-dependent mechanism^22^,^23^,^24^,^25^. A recent study also showed that Munc13-1, the downstream target of the calcium/calmodulin signaling pathway, controls synaptic vesicle replenishment^45^. However, these studies still could not explain our observation of a decrease in the amplitude, but not the time constant, of replenishment_rapid_ with higher calcium influx during more intense stimulation (Figs. 1D, F). Accordingly, our three-pool model solves this problem by adding a small IP, which is responsible for the rapid and slow components of the RRP replenishment time course. The IP controls the amplitude of the replenishment_rapid_. More intense stimulation caused greater depletion of the IP, which decreased the replenishment_rapid_ amplitude. By ruling out the possibility that rapid RRP replenishment is provided by vesicles made from rapid endocytosis or from a small recycling pool, we conclude that the RRP is replenished from the IP. Although currently more detailed characteristics of this pool remain unclear, a recent study showed that in synapsin triple knock-out mice, the RRP replenishment was significantly slowed down and the number of synaptic vesicles distally from the active zones was strongly decreased, whereas those localised at the active zones remained unchanged^46^. Furthermore, most of the synapsin-defined vesicle pool in that study presented as a part of the traditional RP, comprising ~95% of the total synaptic vesicles^18^,^47^,^48^. Another report using synapsin I/II double knock-out mice proposed a local reserve pool three times the size of RRP at hippocampus^49^, which is also very similar to our conclusion. Morphological evidence from EM also showed that synaptic vesicles could be interconnected by synapsin at a distance from AZ^50^. Our result is consistent with all these findings, and we further include endocytosis to give a more complete vesicle recycling model. It would be of great interest to examine whether synapsin is the key molecular entity that can differentiate the IP from the RP and RRP at calyces in the future^18^.

A previous study showing that blocking calmodulin function slowed down endocytosis and RRP replenishment suggested that slowing down of RRP replenishment under stronger stimulation might be caused by occlusion of the active zone by proteins participating in exocytosis^6^,^51^. However, a recent report showed that blocking PKA slowed down endocytosis, but did not affect the recruitment of synaptic vesicles to the RRP^52^. Furthermore, under mild stimulation, blocking calmodulin did not block endocytosis but still slowed down RRP replenishment, both of which suggest independent mechanisms for RRP replenishment and endocytosis^52^. Although our three-pool model was developed for stimulation stronger than a 20 ms depolarisation, it may not necessarily be in conflict with the finding of a calcium/calmodulin-dependent acceleration in the RRP replenishment^25^,^53^, as the later mechanism could be saturated by the calcium influx during a single 20 ms depolarisation. Previous studies showed that a 10–20 ms depolarisation pulse can induce enough calcium influx to deplete the RRP at the calyx of Held synapse^7^,^26^. In such a case, the slowing of RRP replenishment is dominated by the decrease in IP size. For stimulation milder than a 20 ms depolarisation, our model may be modified to include the calcium effect so that the IP-RRP rate constant (k_1_ and k_−1_) or the IP size is calcium/calmodulin-dependent.

Bi-exponential recovery from STD has been observed in many synapses^13^,^22^,^35^,^41^,^42^ where our model may also be helpful. For example, at frog auditory hair cell synapses, recovery draws mainly from the preformed vesicles rather than the rapid, freshly endocytosed vesicles, which is consistent with our model^41^. At hippocampal synapses, several new findings, such as the synapsin-mediated vesicle interconnection^50^ and local reserve pool model^49^, also imply the potential usefulness of our model.

Although our three-pool model was proposed from RRP replenishment, it could also be used to accurately dissect the exocytosis at each time step during stimulation (Figs. 2D–F). Furthermore, we evaluated the impact of endocytosis using our three-pool model and concluded that endocytosis is important in maintaining synaptic transmission during high frequency stimulation, which often happens in the central nervous system. Thus, we concluded that the three-pool model is a useful tool for revealing the contribution of each vesicle cycling step in the maintenance of synaptic transmission, and the generation and recovery of STD during repetitive stimulation.

## Author Contributions

J.G. and L.X. designed research; J.-L.G., M.H. and X.-S.W. performed experiments; J.G., Z.-C.S. and L.X. built the model; J.-B. Z., W.W. and P.-T.Y helped with experiments; W.L. helped verify the model and L.X. supervised the project and wrote the paper.

## Supplementary Material

Supplementary Information Supplementary information
