# Erratum: A Three-Pool Model Dissecting Readily Releasable Pool Replenishment at the Calyx of Held

**DOI:** 10.1038/srep31277

**Published:** 2016-08-25

**Authors:** Jun Guo, Jian-long Ge, Mei Hao, Zhi-cheng Sun, Xin-sheng Wu, Jian-bing Zhu, Wei Wang, Pan-tong Yao, Wei Lin, Lei Xue

Scientific Reports 5: Article number: 9517; 10.1038/srep09517published online: 03 31 2015; updated: 08 25 2016

In the original version of this Article, there were errors in Affiliation 1 which was incorrectly listed as ‘State Key Laboratory of Genetic Engineering, Collaborative Innovation Center of Genetics and Development, Department of Physiology and Biophysics, School of Life Sciences, Fudan University, Shanghai, P.R. China, 200433’. The correct affiliation is listed below:

State Key Laboratory of Medical Neurobiology, Department of Physiology and Biophysics, School of Life Sciences and Collaborative Innovation Centre for Brain Science, Fudan University, Shanghai, 200438, P.R. China.

This error has now been corrected in the HTML version of this Article.

There are errors in Scheme (1),


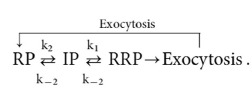


should read:


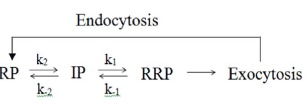


There are errors in Scheme (2),
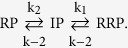


should read:


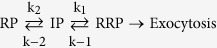


There are errors in Scheme (6),
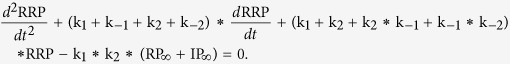


should read:
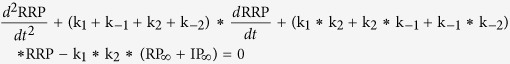


There are errors in Scheme (10)
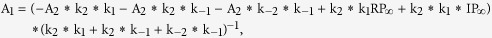


should read:
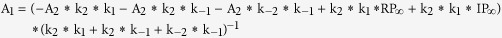


In the Results section under subheading ‘The rate of the RRP replenishment was slower after more intense stimulation’,

“At various times (Δt = 0.05–20 s) after a conditioning 20 ms depolarisation (−80 to +10 mV, if not mentioned), which depleted the RRP (459 ± 29 fF, n = 11), we applied a 20 ms depolarisation to measure the resulting capacitance jump (ΔCm), which reflected the recovery of the RRP (Fig. 1A).”

should read:

“At various times (Δt = 0.05–20 s) after a conditioning 20 ms depolarisation (−80 to +10 mV, if not mentioned), which depleted the RRP (459 ± 29 fF, from ref. 21), we applied a 20 ms depolarisation to measure the resulting capacitance jump (ΔCm), which reflected the recovery of the RRP (Fig. 1B).”

In the Results section under subheading ‘Rapid and slow vesicle traffic among three pools underlie rapid and slow RRP replenishment’,

“The above parameters were obtained by comparing the model with the observed RRP replenishment after a single 20 ms depolarization (Fig. 2A, black curve).”

should read

“The above parameters were obtained by comparing the model with the observed RRP replenishment after a single 20 ms depolarization (Fig. 2A).”

In the same section,

“The predicted total exocytosis amount (with endo: 2.3 ± 0.1 pF, without endo: 2.2 ± 0.1, n = 6) also closely matched the measured net exocytosis (2.3 ± 0.1 pF, n = 6, p = 0.8 with endocytosis, Fig. 2F).”

should read:

“The predicted total exocytosis amount (with endo: 2.3 ± 0.1 pF, without endo: 2.2 ± 0.1 pF, n = 6) also closely matched the measured net exocytosis (2.3 ± 0.1 pF, n = 6, p = 0.8 with endocytosis, Fig. 2F).”

The Acknowledgements section is incomplete,

“This work was sponsored by Shanghai Pujiang Program, National Natural Science Foundation of China (grant number: 31370828), Specialised Research Fund for the Doctoral Program of Higher Education (SRFDPF, grant number: 20120071120013) and the Shanghai Leading Academic Discipline Project (B111).”

should read:

“This work was sponsored by Shanghai Pujiang Program, National Natural Science Foundation of China (grant number: 31370828), National High-tech R&D Program of China (2015AA020512), Specialised Research Fund for the Doctoral Program of Higher Education (SRFDPF, grant number: 20120071120013) and the Shanghai Leading Academic Discipline Project (B111).”

There is a typographical error in the Author Contributions statement,

“J.-B. Z., W.W. and P.-t.Y. helped with experiments”

should read:

““J.-B. Z., W.W. and P.-T.Y. helped with experiments”

