# Supplementary material for: A Three-Pool Model Dissecting Readily Releasable Pool Replenishment at the Calyx of Held
Source: Sci Rep. 2015 Mar 31;5:9517. doi: 10.1038/srep09517 (PMC4379469; doi:10.1038/srep09517)
Supplement: Supplementary Information [file srep09517-s1.doc]

**A Three-Pool Model Dissecting Readily Releasable Pool Replenishment at the Calyx of Held**

Jun Guo^1#^, Jian-long Ge^1#^, Mei Hao^1#^, Zhi-cheng Sun^1#^, Xin-sheng Wu^2^, Jian-bing Zhu^1^, Wei Wang^1^, Pan-tong Yao^1^, Wei Lin^3^ and Lei Xue^1*^

1. State Key Laboratory of Genetic Engineering, Collaborative Innovation Center of Genetics and Development, Department of Physiology and Biophysics, School of Life Sciences, Fudan University, Shanghai, P.R. China, 200433

2. Synaptic Transmission Section, National Institute of Neurological Disorders and Stroke, Maryland, 20892

3. School of Mathematical Sciences, Centre for Computational Systems Biology and Shanghai Centre for Mathematical Sciences, Fudan University, P.R.China, 200433

#: These authors contribute equally to this work.

*: Corresponding author

**Supplementary Information**

1. **RRP replenishment accelerates at physiological temperature**

High temperatures lead to accelerated endocytosis and faster recovery from synaptic depression at calyces^1^. To test whether our model can be applied to the accelerated RRP replenishment at physiological temperature (~ 34°C, PT), we repeated the experiments in Fig. 1 at PT. Our results confirmed that RRP replenishment was dramatically accelerated after a single 20 ms depolarisation or 10 pulses of 20 ms depolarisation at 1–10 Hz (Figs. S1A-C). To better predict RRP replenishment at PT, we modified our model by multiplying each reaction rate by a new ratio constant (h, see scheme S1). When h falls in the range of 2–4, the predicted results still match well with the measurements at PT, which further strengthens our model.


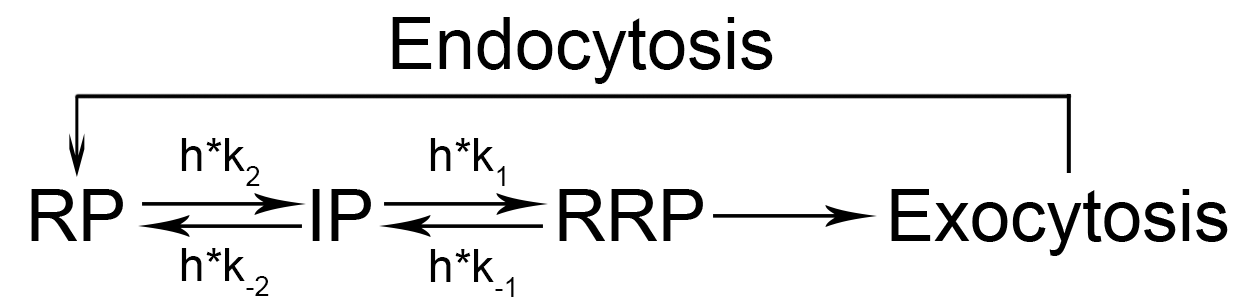
 (S1)


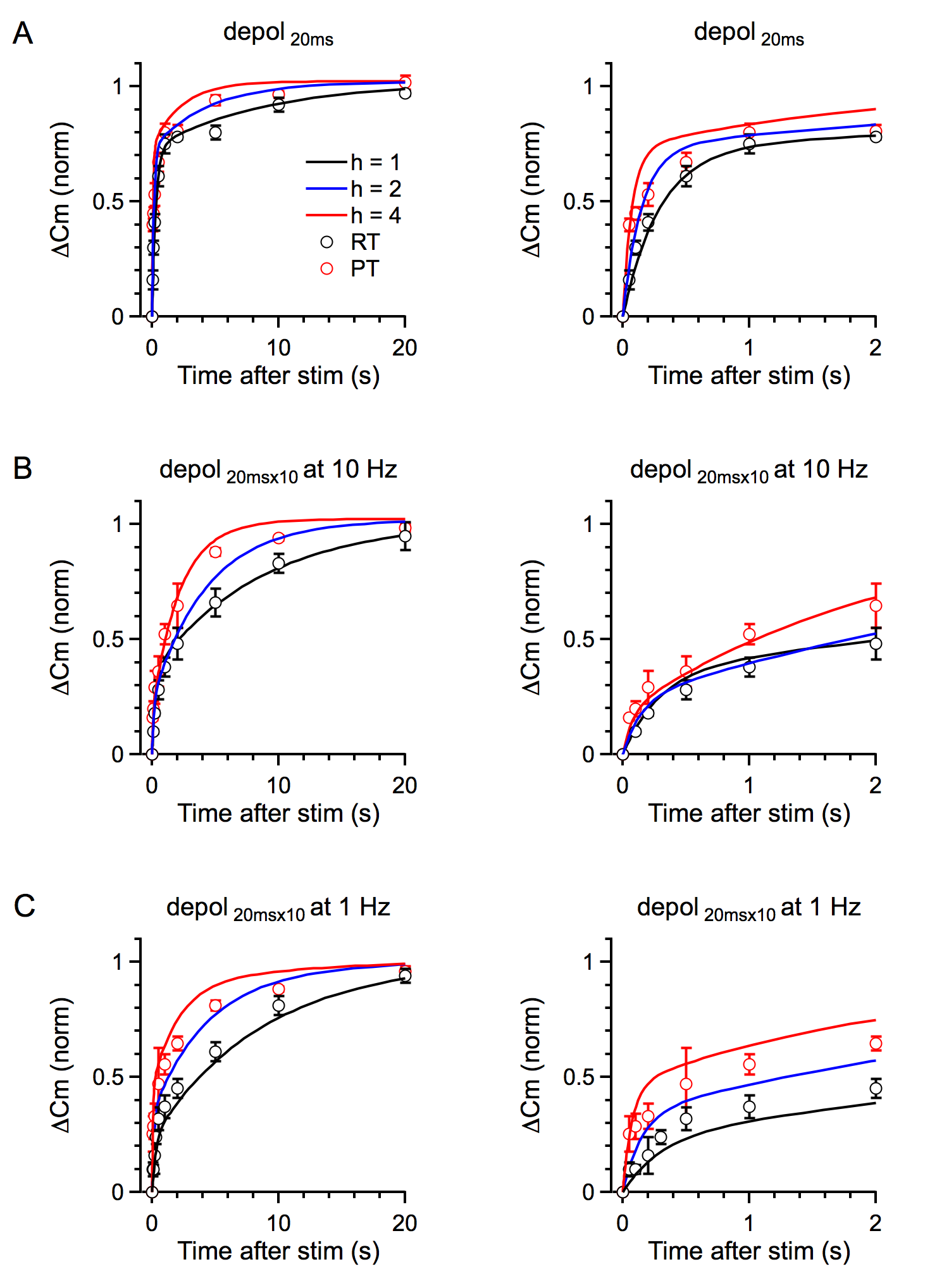


Figure S1. Accelerated RRP replenishment at physiological temperature

1. Left: Cm induced by a 20 ms depolarisation applied at various intervals after a conditioning 20 ms depolarisation at PT (n = 5, red circle). Data collected at room temperature (RT, black circle, same as in Fig. 1B) and the model-predicted RRP replenishment curves with different h values are shown for comparison (see scheme S1, black curve, h = 1; blue curve, h = 2; red curve, h = 4). Right: Same data as in the left panel but shown at a different time scale.
2. Similar to A, but with a conditioning stimulus of 10 pulses of 20 ms depolarisation at 10 Hz (n = 4).
3. Similar to A, but with a conditioning stimulus of 10 pulses of 20 ms depolarisation at 1 Hz (n = 3).
4. **Accelerated endocytosis does not affect RRP replenishment at physiological temperatures**

In the present study, we have shown that endocytosis recycles vesicles into the RP and does not affect RRP replenishment. However, physiological temperature dramatically accelerates vesicle endocytosis^2^. Recently, an ultra-fast endocytosis was reported at hippocampal synapses^3^. Here we further tested whether these forms of accelerated endocytosis will influence our conclusion at RT (Fig. S2A) and at PT (Fig. S2B). Renden et al. reported a fast component of endocytosis (46%) with a time constant of 2.2 s and a slow component of endocytosis (54%) with a time constant of 13 s^2^. For the ultra-fast endocytosis, it peaks at 100 ms after stimulation and retrieves 85% of the total exocytosed vesicles. Thus, we made the simulation composed of a fast component (85%) with a time constant of 100 ms and a slow component (15%) with a time constant of 15 s. The model-predicted RRP replenishment was shown in Fig. S2. At RT, these two accelerated endocytosis do not affect RRP replenishment (Fig. S2A). Even when the reaction constants are multiplied by a ratio of two (h = 2, see scheme S1) to mimic the RRP replenishment at PT, these two accelerated endocytosis still do not contribute much to the RRP replenishment (Fig. S2B). Therefore, the accelerated endocytosis does not affect our conclusion.


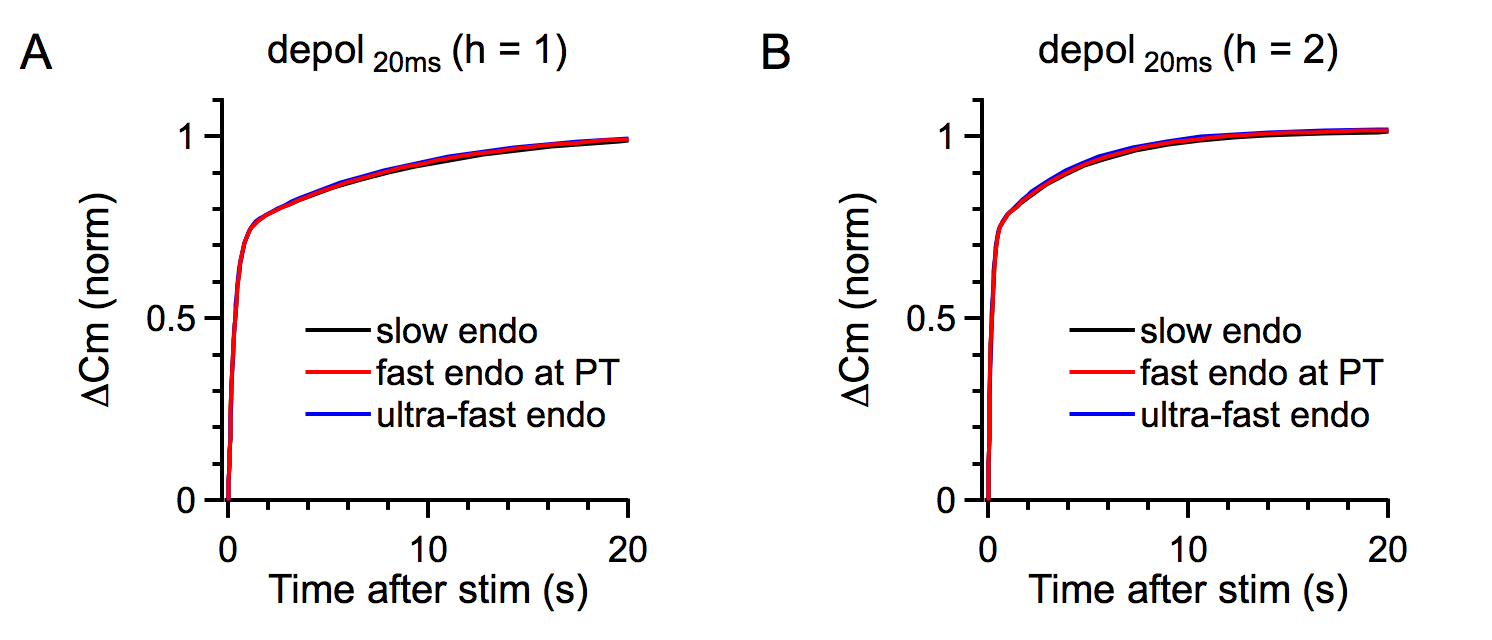


Figure S2. Accelerated endocytosis or ultra-fast endocytosis does not affect RRP replenishment

1. Model-predicted RRP replenishment curve with endocytosis rate at room temperature (black, 100% slow endocytosis, τ = 15 s) or physiological temperature (red, 46% fast endocytosis with τ = 2.2 s and 54% slow endocytosis with τ = 13 s; blue, 85% ultra-fast endocytosis with τ = 100 ms and and 15% slow endocytosis with τ = 15 s).
2. Similar to A, except that the h value is set to 2 to mimic the physiological temperature.

References

1 Kushmerick, C., Renden, R. & von Gersdorff, H. Physiological temperatures reduce the rate of vesicle pool depletion and short-term depression via an acceleration of vesicle recruitment. *Journal of Neuroscience* **26**, 1366-1377, doi:10.1523/jneurosci.3889-05.2006 (2006).

2 Renden, R. & von, G. H. Synaptic vesicle endocytosis at a CNS nerve terminal: faster kinetics at physiological temperatures and increased endocytotic capacity during maturation. *J.Neurophysiol.* **98**, 3349-3359 (2007).

3 Watanabe, S. *et al.* Ultrafast endocytosis at mouse hippocampal synapses. *Nature* **504**, 242-247, doi:10.1038/nature12809 (2013).
